# Phenotypic characterization of Pomeranians with or without Chiari-like malformation and syringomyelia

**DOI:** 10.3389/fvets.2023.1320942

**Published:** 2023-12-19

**Authors:** Koen M. Santifort, Ines Carrera, Kenny Bossens, Paul J. J. Mandigers

**Affiliations:** ^1^Neurology, IVC Evidensia Referral Hospital Arnhem, Arnhem, Netherlands; ^2^Neurology, IVC Evidensia Referral Hospital Hart van Brabant, Waalwijk, Netherlands; ^3^Expertise Centre of Genetics, Department of Clinical Sciences, Faculty of Veterinary Medicine, Utrecht University, Utrecht, Netherlands; ^4^Vet Oracle Teleradiology, Norfolk, United Kingdom; ^5^Department of Neurology, Orion Small Animal Hospital, Herentals, Belgium

**Keywords:** magnetic resonance imaging, syrinx, central canal dilatation, screening, welfare

## Abstract

**Introduction:**

Chiari-like malformation (CM) and syringomyelia (SM) are frequently diagnosed conditions in small and toy dog breeds, such as the Cavalier King Charles Spaniel and Griffon Bruxellois. CM/SM is only rarely reported in Pomeranians in literature to date. The aims of this study are to 1/describe the phenotype of Pomeranians with or without CM/SM and 2/evaluate for differences and associations between CM/SM and owner-reported clinical signs (ORCS) or signalment factors.

**Materials and methods:**

From February 2015 to June 2023, historical data and signalment (including country of origin, pedigree, sex and neuter status, age, and body weight) and ORCS of Pomeranians were recorded at multiple institutions. MRI studies of all dogs were evaluated for classification of CM/SM. Additionally, quantitative measurements were performed for SM.

**Results:**

A total of 796 dogs from 22 different countries were included. Total prevalence of CM was 54.9% (437/796) and the prevalence of SM was 23.9% (190/796). The top 5 ORCS included 1/scratching with skin contact, rubbing head or ears, or both (57.6% of dogs with ORCS), 2/air licking (30.7% of dogs with ORCS), 3/spontaneous signs of pain (26.0% of dogs with ORCS), 4/persistent licking front and/or hind paws (22.6% of dogs with ORCS), 5/phantom scratching (22.6% of dogs with ORCS). Phantom scratching, vocalization, head shaking, spontaneous signs of pain, and air licking were associated with having SM. There were no statistically significant associations between quantitative syrinx measurements and ORCS. There were statistically significant associations between CM classification and 1/country of origin, 2/having a pedigree, and 3/age. There were statistically significant associations between SM classification and 1/age and 2/body weight.

**Discussion:**

This is the first large study evaluating CM/SM in the Pomeranian dog breed. Veterinary clinicians can use these findings to increase the likelihood of correctly determining the presence or absence of CM/SM in Pomeranians. Breeders may consider using the information regarding signalment factors as well as ORCS associated with CM/SM classifications to select dogs for screening procedures. But an MRI-based diagnosis is needed to properly ascertain the exact CM/SM status of their breeding stock until a fool-proof characteristic or genetic marker is found.

## Introduction

Chiari-like malformation (CM) and syringomyelia (SM) are frequently diagnosed conditions in small and toy dog breeds, such as the Cavalier King Charles Spaniel (CKCS) and Griffon Bruxellois (1–5). The term CM is often employed in veterinary literature to describe a complex malformation of the (caudal cranial fossa of the) skull. Syringomyelia (SM) is the accumulation of fluid in a cavity in the spinal cord, a so-called syrinx (1–5). The term “central canal dilation” (CCD) is sometimes used interchangeably, while some only use this term to refer to a syrinx <2 mm in diameter. Although numerous publications have contributed to improved understanding of these disorders in dogs, especially regarding the CKCS, its pathogenesis is still incompletely understood (1, 3, 4, 6–11).

As CM prevalence in the CKCS is very high (up to 100%), clinical signs related to CM are difficult to establish since there are no or only very few ‘healthy controls' within the breed (1, 3, 8, 12, 13). SM has a variable prevalence within the CKCS, Griffon Bruxellois, and other dog breeds. Depending on the studied population, SM prevalence can be very high (up to 70%) (1, 3, 8, 9, 12–19). A large number of clinical signs and owner-reported clinical signs (ORCS) have been described in literature in dogs with CM/SM (1, 3, 9, 10, 12, 15–19). Some of these have been related to SM dimensions and/or location and have been interpreted as signs of (neuropathic) pain (1, 3, 9, 10, 12, 15, 18–20). The term CM associated pain (CM-P) has been applied to dogs without SM but with CM that show signs of pain (10, 11, 18, 19).

Most of the ORCS are not specific to either of these conditions on their own. Moreover, interpreting them as indicative of pain is inherently subjective. Indeed, relying on owners to report signs has limitations. However, it is of great importance to evaluate the value of ORCS for the diagnosis of CM/SM. Without knowing what ORCS are related to CM/SM, clinicians cannot assess whether dogs with ORCS are likely to have these disorders or not. As diagnostic procedures such as magnetic resonance imaging (MRI) studies require general anesthesia to be performed and costs are incurred by owners for such procedures, knowledge of which ORCS linked to CM/SM could help owners and clinicians to ascertain the need for an MRI.

Although there are only a few documented cases of Pomeranians with CM/SM in veterinary literature, owners, breed clubs and veterinarians realize that CM/SM does occur with Pomeranians (1, 3, 19–22). In 2014, an owner of eight Pomeranians, referred herself to the last author (PM) as all eight dogs showed clinical signs reported as suggestive for CM/SM. All eight Pomeranians were diagnosed with either CM, SM or CM/SM. In 2015, a call was placed on social media platforms by this owner and the Belgian and Dutch breed clubs, to present their Pomeranians for screening by MRI to estimate the prevalence of these two disorders in this breed. Some owners had observed signs, some had not but were worried that their dog could be affected.

The aims of this study are to (1) describe the phenotype of Pomeranians with or without CM/SM and (2) evaluate for differences and associations between CM/SM and ORCS or signalment factors. Our hypotheses are that:

1) there are no statistically significant associations between the classification of CM/SM and ORCS.
2) there are no statistically significant associations between quantitative syrinx measurements and ORCS.
3) there are no statistically significant associations between signalment and classification of CM and/or SM.

## Materials and methods

During the period of February 2015 to June 2023, Pomeranians, with or without a pedigree, with or without ORCS, were presented to multiple institutions for a so-called screening MRI study for CM/SM (17). All owners agreed to participate in this study and an informed consent of the owner was obtained. Signalment factors including country of origin, pedigree, sex and neuter status, age, and body weight were recorded. According to a standardized form, historical data pertaining to the presence or absence of ORCS was acquired via questioning of the owners during consultation and/or owners were requested to fill in an online questionnaire. MRI studies were performed under general anesthesia (individualized anesthetic protocols) with either a low-field MRI scanner (<0.5T MRI) or a high-field MRI scanner (1.5T MRI) depending on the institution involved. The majority of scans were made at four institutions: (1) Department of Clinical Sciences, Utrecht University, The Netherlands (high-field MRI scanner), (2) IVC Evidensia Referral Hospital Arnhem, The Netherlands (high-field MRI scanner), (3) Dierenkliniek den Heuvel, Best, The Netherlands (low-field MRI scanner) and (4) Orion Clinic, Herentals, Belgium (low-field MRI scanner). Sequences obtained included a minimum of sagittal T2-weighted (T2W), sagittal T1W and transverse T2W or T1W sequences of the craniocervicothoracic region. Inclusion of the thoracic spinal cord up to at least the 4th thoracic vertebra was required. Measurements were performed with use of imaging software (Radiant DICOM viewer) by the first author (KS).

### Exclusion criteria

Dogs diagnosed with intracranial space-occupying lesions or space-occupying lesions in the vertebral canal were excluded. MRI studies with artifacts or insufficient image quality that did not allow for accurate assessments or measurements were also excluded.

### Classification of Chiari-like malformation (CM)

**Figure 1:**
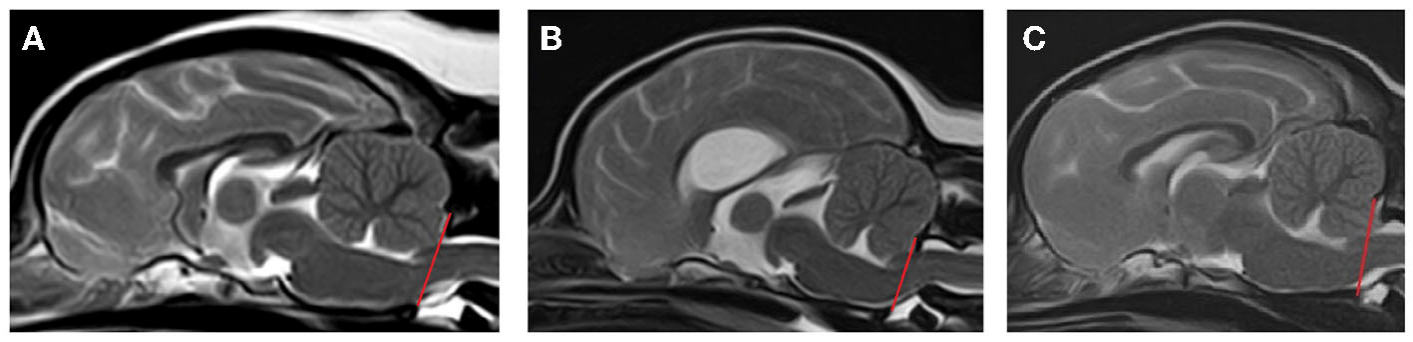
Classification of Chiari-like malformation (CM). **(A)** CM0 = normal—no cerebellar herniation or impaction (cerebellar uvula rostral to foramen magnum), **(B)** CM1 = abnormal—cerebellar impaction (cerebellar uvula on the line of the foramen magnum, no CSF present dorsal to the cervicomedullary junction) and non-rounded shape (e.g., flattened, pointed or indented by supraoccipital bone), **(C)** CM2 = abnormal—cerebellar herniation (cerebellar uvula caudal to the line of the foramen magnum, no CSF present dorsal to the cervicomedullary junction) and non-rounded shape (e.g., pointed or indented by supraoccipital bone).

Images were evaluated to assess the presence or absence of CM by evaluating shape of the cerebellum and position of the caudoventral cerebellum (uvula). CM was classified as follows (Figure 1)^1^:

1. CM0 normal—no cerebellar herniation or impaction (cerebellar uvula rostral to foramen magnum).
2. CM1 abnormal—cerebellar impaction (cerebellar uvula on the line of the foramen magnum, no CSF present dorsal to the cervicomedullary junction) and non-rounded shape (e.g., flattened, pointed or indented by supraoccipital bone).
3. CM2 abnormal—cerebellar herniation (cerebellar uvula caudal to the line of the foramen magnum, no CSF present dorsal to the cervicomedullary junction).
  CM normal = CM0, CM abnormal = CM1 and CM2.
  The line of the foramen magnum was defined as a straight line between the most ventral aspect of the supraoccipital bone and the most caudal aspect of the basioccipital bone on sagittal MR images (23).

### Classification of syringomyelia (SM)

**Figure 2:**
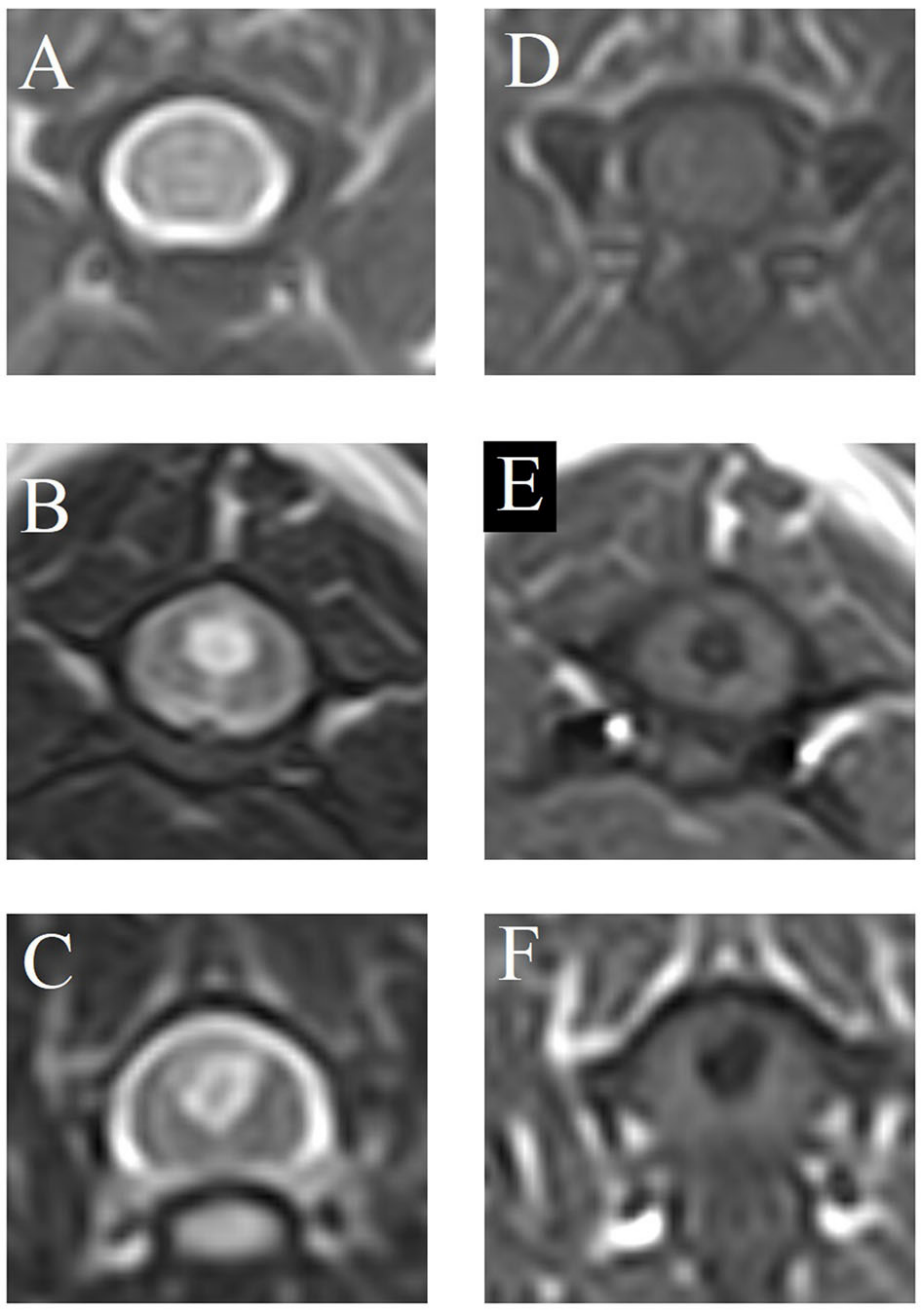
Classification of syringomyelia (SM). **(A, D)** SM0 = normal—no SM, **(B, E)** SM1 = abnormal—symmetric (i.e., circular, round syrinx), **(C, F)** SM2 = abnormal—asymmetric (e.g., syrinx extending into a dorsal horn). **(A–C)** T2-weighted magnetic resonance images, transverse. **(D–F)** T1-weighted magnetic resonance images, transverse.

Images were evaluated to assess the presence or absence of SM in the spinal cord. SM was defined as a well-demarcated intramedullary cavity associated with the central canal of the spinal cord, hyperintense on T2W and hypointense on T1W images. SM was classified as follows (Figure 2):

1. SM0 normal—no SM.
2. SM1 abnormal—symmetric (i.e. circular, round syrinx).
3. SM2 abnormal—asymmetric (e.g. syrinx extending into a dorsal horn).

SM normal = SM0, SM abnormal = SM1 and SM2

**Figure 3:**
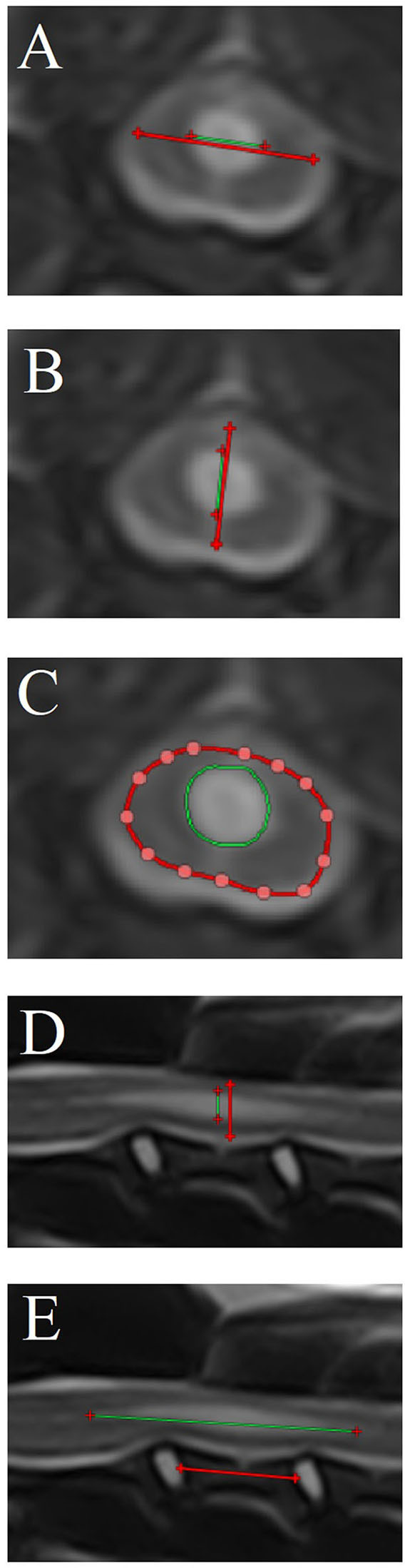
Quantitative syrinx measurements (only T2-weighted magnetic resonance images are shown). **(A)** Maximum transverse syrinx width/spinal cord width ratio [STWR—T2W (and T1W when available) transverse images], **(B)** Maximum syrinx height/spinal cord height ratio on transverse images [SHRt—T2W (and T1W when available) transverse images]. **(C)** Maximum syrinx cross-sectional area/spinal cord cross-sectional area ratio [SCSAR – T2W (and T1W when available) transverse images], **(D)** Maximum syrinx height/spinal cord height ratio on sagittal images (SHRs—T2W and T1W sagittal images), **(E)** Length of the syrinx/C3 vertebral body length ratio (SLC3R—T2W and T1W sagittal images). Syrinx parameter measurements are depicted as green lines **(A, B, D, E)** or cross sectional areas **(C)**. Reference measurements [spinal cord in **(A–D)**—C3 vertebra in **(E)**] are depicted as red lines **(A, B, D, E)** or cross sectional areas **(C)**.

Quantitative measurements were performed when a syrinx was present, including (Figure 3):

- Maximum transverse syrinx width/spinal cord width ratio (STWR—T2W and T1W transverse images).
- Maximum syrinx height/spinal cord height ratio on transverse images (SHRt—T2W and T1W transverse images).
- Maximum syrinx cross-sectional area/spinal cord cross-sectional area ratio (SCSAR—T2W and T1W transverse images).
- Maximum syrinx height/spinal cord height ratio on sagittal images (SHRs—T2W and T1W sagittal images).
- Length of the syrinx:C3 vertebral body length ratio (SLC3R—T2W and T1W sagittal images).

Additionally, it was noted where the syrinx was localized: cervical, thoracic, extensive (both cervical and thoracic, continuous), or multifocal (both cervical and thoracic, discontinuous). For extensive and multifocal localizations, the most severely affected location (cervical or thoracic) was noted based on maximum SHRs. Length of the syrinx was not measured for extensive of multifocal localizations.

### Classification based on owner-reported clinical signs

Dogs were classified as “without” ORCS or “with” ORCS. Specific ORCS were recorded as present or absent (see **Table 4** for specific ORCS).

Table 1 summarizes the classification of dogs according to CM, SM, and ORCS.

**Table 1:** Classification of dogs based on MRI evaluations and owner-reported clinical signs.

**CM classification**	
CM normal	No cerebellar herniation or impaction (cerebellar uvula rostral to foramen magnum)
CM abnormal	CM abnormal 1: cerebellar impaction (cerebellar uvula on the line of the foramen magnum, no CSF present dorsal to the cervicomedullary junction) and non-rounded shape (e.g., flattened, pointed or indented by supraoccipital bone). CM abnormal 2: cerebellar herniation (cerebellar uvula caudal to the line of the foramen magnum, no CSF present dorsal to the cervicomedullary junction) and non-rounded shape (e.g., pointed or indented by supraoccipital bone). The line of the foramen magnum was defined as a straight line between the most ventral aspect of the supraoccipital bone and the most caudal aspect of the basioccipital bone on sagittal MR images (23).
**SM classification**	
SM normal	No SM
SM abnormal	SM abnormal 1: symmetric (i.e. circular, round syrinx). SM abnormal 2: asymmetric (e.g., extending into a dorsal horn). Localization: cervical, thoracic, extensive (both cervical and thoracic, continuous), or multifocal (both cervical and thoracic, discontinuous). For extensive and multifocal localizations, the most severely affected location (cervical or thoracic) was noted (C:x–T:x) based on max. SHRs.
**ORCS classification**	
Without ORCS	No ORCS
With ORCS	ORCS present

CM, Chiari-like malformation; CSF, cerebrospinal fluid; SM, syringomyelia; ORCS, owner-reported clinical signs; max. SCSAR, maximum syrinx cross-sectional area:spinal cord cross-sectional area ratio.

### Statistical analysis

Descriptive statistics are reported. A Kolmogorov-Smirnov test was used to assess if the data followed a normal distribution. Differences between groups and associations between the presence of ORCS and signalment factors, CM classification, SM classification, SM localization, and quantitative syrinx measurements were analyzed by making use of a Mann-Whitney U test, Fisher's exact test, and Chi-squared test of independence where appropriate. Odds ratios were calculated for significant results and reported with a 95% confidence interval (OR, 95% CI). *P* < 0.05 were regarded as significant. Correlations between T2W-based and T1W-based measurements were assessed by calculation of the Pearson correlation coefficient (*r*, 95% CI). Analyses were performed using Microsoft Excel and R v4.3.1.

## Results

### Descriptive statistics

A total of 871 Pomeranians were identified, of which 796 met the inclusion criteria. Table 2 includes the descriptive statistics regarding the study population. The 796 dogs originated from 22 different countries, with the majority (556; 69.9%) originating from either Russia (304; 38.2%) or The Netherlands (252; 31.7%). The origin of 114 (14.3%) dogs was unknown. Five hundred and thirty-one (531; 66.7%) dogs were pure-bred dogs and 265 (33.3%) were not (i.e., proper documentation was lacking or the dog was not registered at any national kennel club). The male:female ratio was 0.98:1.00, with 395 (49.6%) males of which 15 (3.8%) were neutered and 401 (50.4%) females of which 13 (3.2%) were neutered. Median age was 2.19 years (range 0.17–11.02). Body weight of 385 dogs (48.4%) was available for analysis. Median body weight was 3.3 kg (range 1.0–9.4).

**Table 2:** Descriptive statistics of the study population.

Total study population	796 (100%)
**Country of origin**	**796 (100%)**
Netherlands	252 (31.7%)
Russia	304 (38.2%)
Other EU	72 (9,0%)
Other non-EU	54 (6,8%)
Unknown	114 (14.3%)
**Pedigree**	**796 (100%)**
Yes	531 (66.7%)
No	265 (33.3%)
**Sex**	**796 (100%)**
- Male	395 (49.6%) Male intact: 380 (96.2%) Male neutered: 15 (3.8%)
- Female	401 (50.4%) Female intact: 388 (96.8%) Female neutered: 13 (3.2%)
**Age**	**796 (100%)**
	Median 2.19 years (range 0.17–11.02)
**Weight**	**385 (48.4%)**
	Median 3.3 kg (range 1.0–9.4)

Other EU, other European Union countries, including Belgium, Bulgaria, Czech Republic, Estonia, France, Germany, Hungary, Italy, Latvia, Poland, Romania, and Spain. Other non-EU, other non-European Union countries, including Belarus, Brasil, Kyrgyzstan, Mexico, Norway, Ukraine, Turkey, and the United States of America.

The prevalence of classifications of CM and SM are included in a contingency table (Table 3). The total prevalence of CM/SM Pomeranians included in this study, the prevalence of CM/SM in Pomeranians with ORCS (i.e., those presented for specific evaluation for the presence or absence of CM/SM = 418/796; 52.5% of the total study population), and the prevalence of CM/SM in Pomeranians without ORCS (i.e. those presented for screening purposes: 378/796; 47.5% of the total study population) are included here:

- Total prevalence of CM was 54.9% (437/796). Total prevalence of SM was 23.9% (190/796).
- Prevalence of CM in dogs with ORCS was 62.4% (261/418). Prevalence of SM in dogs with ORCS was 34.7% (145/418).
- Prevalence of CM in dogs without ORCS was 53.4% (202/378). Prevalence of SM in dogs without ORCS was 11.9% (45/378).

**Table 3:** Contingency table including numbers and percentages of dogs combining CM and SM classifications.

**Classification**	**SM 0**	**SM 1**	**SM 2**	**Total**
CM 0	327 (41.1%)	25 (3.1%)	7 (0.9%)	359 (45.1%)
CM 1	244 (30.7%)	117 (14.7%)	27 (3.4%)	388 (48.7%)
CM 2	35 (4.4%)	9 (1.1%)	5 (0.6%)	49 (6.2%)
Total	606 (76.1%)	151 (19.0%)	39 (4.9%)	796 (100%)

CM, Chiari-like malformation; SM, syringomyelia.

Localization of SM was multifocal for 89/190 dogs (46.8%), cervical for 77/190 dogs (40.5%), and extensive for 24/190 dogs (12.6%). In the multifocal and extensive SM cases, 47/89 (52.8%) and 16/24 (66.7%) had more severe SM in the thoracic spinal cord than the cervical spinal cord, respectively.

ORCS are included in Table 4. A total of 376/796 (47.2%) dogs did not have ORCS and 420/796 (52.8%) did have ORCS. The top 5 ORCS included (1) scratching with skin contact, rubbing head or ears, or both (57.6% of dogs with ORCS), (2) air licking (30.7% of dogs with ORCS), (3) spontaneous signs of pain (26.0% of dogs with ORCS), (4) persistent licking front and/or hind paws (22.6% of dogs with ORCS), (5) phantom scratching (22.6% of dogs with ORCS).

**Table 4:** Owner-reported clinical signs (ORCS) including number and percentage of dogs with specific ORCS.

**ORCS classification**	**796 (100%)**	**Explanation**
Without ORCS	376 (47.2%)	No ORCS
With ORCS	420 (52.8%)	ORCS present (any number of ORCS)
**Specific ORCS**	(100%)	
1. Scratching with skin contact, rubbing head or ears, or both	242 (57.6%)	Scratching head, neck or shoulder areas with front or hind limbs. Rubbing head or ears with front limbs, on walls, or on the floor.
2. Air licking	129 (30.7%)	Licking the air frequently and repetitively.
3. Spontaneous signs of pain	109 (26.0%)	Any sign that suggested to the owner the dog may be or have been experiencing pain that is not elicited by external stimuli or activities (e.g., touch).
4. Persistent licking front and/or hind paws	95 (22.6%)	Excessively and/or repetitively licking front and/or hind limbs without identifiable skin disease.
5. Phantom scratching	95 (22.6%)	Scratching toward the neck area, but not making skin-contact.
6. Provoked signs of pain	62 (14.8%)	Any sign that suggested to the owner the dog may be or have been experiencing pain that was elicited by external stimuli or activities [e.g., by being picked up, touched, or when asked to perform certain activities (playing)].
7. Head shaking	60 (14.3%)	Unprovoked head shaking.
8. Lethargy	57 (13.6%)	E.g. increased sleepiness, decreased excitement.
9. Hyperexcitability	56 (13.3%)	E.g. overreaction to external stimuli, inability to relax, episodically running in circles.
10. Fly catching or tail chasing	54 (12.9%)	Repetitively, episodically biting the air (as if there is a fly to catch) or chasing the tail.
11. Vocalization	39 (9.3%)	Yelping, yelling, screaming, barking (without identifiable other reasons).
12. Aggression	28 (6.7%)	Toward other dogs, companion animals, house mates, visitors, or owners.
13. Swallowing, yawning, panting	20 (4.7%)	Excessive or repetitive swallowing unassociated with food or water intake, excessive or repetitive yawning, excessive panting unrelated to exercise.
14. Weakness	18 (4.3%)	Tripping, falling, difficulty walking or supporting weight.
15. Facial expression suggestive of pain	12 (2.9%)	Changes in facial expression interpreted by the owner as signs of pain (e.g., squinting eyes, “unhappy” look).

Statistics for quantitative syrinx measurements are included in Table 5. All T1W-based measurements were smaller than T2W-based measurements, meaning that STWR, SHRt, SCSAR, SHRs, and SLC3R were all higher when based on T2W-measurements than when based on T1W-measurements. There was a statistically significant difference between all T2W-based measurements and T1W-based measurements (*p* ≤ 0.0274). T2W-based and T1W-based measurements for STWR, SHRt, SCSAR, SHRs, and SLC3R were all very strongly correlated (*r* ≥ 0.90, *p* < 0.0001).

**Table 5:** Quantitative syrinx measurements of Pomeranians with syringomyelia (SM abnormal dogs).

**Measurement**	**Number of dogs**	**Median**	**Range**	**P-value for differences between T2W-based vs. T1W-based measurements**	**Correlation between T2W-based and T1W-based measurements**
STWR—T2W	173	0.41	0.15–0.88	T1W-based measurement was smaller in 100% of cases. *p* = 0.0011	*r* = 0.92 (*p* < 0.0001)
STWR—T1W	136	0.34	0.08–0.83		
SHRt—T2W	173	0.56	0.18–0.90	T1W-based measurement was smaller in 100% of cases. *p* = 0.0029	*r* = 0.95 (*p* < 0.0001)
SHRt—T1W	136	0.50	0.17–0.84		
SCSAR—T2W	173	0.22	0.04–0.71	T1W-based measurement was smaller in 100% of cases. *p* = 0.0002	*r* = 0.93 (*p* < 0.0001)
SCSAR—T1W	136	0.16	0.02–0.69		
SHRs—T2W	176	0.49	0.15–0.84	T1W-based measurement was smaller in 100% of cases. *p* < 0.0001	*r* = 0.90 (*p* < 0.0001)
SHRs—T1W	162	0.40	0.00–0.72		
SLC3R—T2W	63	2.84	0.33–6.57	T1W-based measurement was smaller in 100% of cases. *p* = 0.0274	*r* = 0.96 (*p* < 0.0001)
SLC3R—T1W	52	2.01	0.27–5.57		

SCSAR, maximum syrinx cross-sectional area/spinal cord cross-sectional area ratio on transverse images, SHRs, maximum syrinx height/spinal cord height ratio on sagittal images, SHRt maximum syrinx height:spinal cord height ratio on transverse images, SLC3r, length of the syrinx/C3 vertebral body length ratio on sagittal images, and STWr, maximum transverse syrinx width/spinal cord width ratio on transverse images.

### Classification of CM and ORCS

There was a significant difference between CM normal (CM0) dogs and CM abnormal dogs (CM1 and CM2) with regard to the presence or absence of ORCS (*p* < 0.0001). CM abnormal dogs were 1.9 times more likely to have ORCS than CM normal dogs (OR 1.9, 95% CI 1.4–2.5). There was no significant difference between CM1 and CM2 dogs (*p* = 0.4181).

Five ORCS occurred significantly more often in CM abnormal dogs compared to CM normal dogs: (1) phantom scratching (OR 4.4, 95% CI 2.5–7.5, *p* < 0.0001), (2) vocalization (OR 2.2, 95% CI 1.1–4.4, *p* = 0.0298), (3) scratching with skin contact, rubbing head or ears, or both (OR 1.8, 95% CI 1.3–2.5, *p* = 0.0002), (4) spontaneous signs of pain (OR 1.6, 95% CI 1.0–2.4, *p* = 0.0354), and (5) air licking (OR 1.5, 95 CI 1.0–2.3, *p* = 0.0308).

There were no specific ORCS that occurred significantly more or less often in CM1 compared to CM2 dogs.

### Classification of SM and ORCS

There was a significant difference between SM normal (SM0) dogs and SM abnormal dogs (SM1 and SM2) with regard to the presence or absence of ORCS (*p* < 0.0001). SM abnormal dogs were 3.9 times more likely to have ORCS than SM normal dogs (OR 3.9, 95% CI 2.7–5.7). There was no significant difference between SM1 and SM2 dogs (*p* = 0.1902).

Localization of SM was not associated with the presence or absence of ORCS (*p* = 0.2500).

Five ORCS occurred significantly more often in SM abnormal dogs compared to SM normal dogs: (1) spontaneous signs of pain (OR 3.6, 95% CI 2.4–5.5, *p* < 0.0001), (2) phantom scratching (OR 3.3, 95% CI 2.1–5.1, *p* < 0.0001), (3) vocalization (OR 3.3, 95% CI 1.7-6.2, *p* = 0.0002), (4) air licking (OR 2.6, 95% CI 1.7–3.9, *p* < 0.0001), and 5/scratching with skin contact, rubbing head or ears, or both (OR 1.8, 95% CI 1.3–2.5, *p* = 0.001).

There were no specific ORCS that occurred significantly more or less often in SM1 compared to SM2 dogs.

### Combined classification of CM and SM, and ORCS

Table 6 includes the odds ratios for having ORCS for comparisons between combined classifications of CM and SM. Dogs classified as either CM or SM abnormal or both increased the likelihood of having ORCS compared to CM and SM normal dogs (OR between 1.5 and 4.9). Dogs having only CM were 1.5 times more likely to have ORCS than CM and SM normal dogs. Dogs having SM were 4.4 times more likely to have ORCS than CM and SM normal dogs, and 3.0 times more likely to have ORCS than CM abnormal/SM normal dogs.

**Table 6:** Comparison between combined classifications of Chiari-like malformation (CM) and syringomyelia (SM).

**Combined classification**	**Compared to**	**OR (95% CI)**	***p*-value**
CM abnormal/SM abnormal	CM normal/SM normal	4.9 (3.2–7.6)	*p* < 0.0001
CM normal/SM abnormal	CM normal/SM normal	4.4 (1.9–10.0)	*p* = 0.0014
CM abnormal/SM normal	CM normal/SM normal	1.5 (1.1–2.0)	*p* = 0.0434

Odds ratios {OR [95% confidence interval (CI)]} for having owner-reported signs (ORCS).

**Figure 4:**
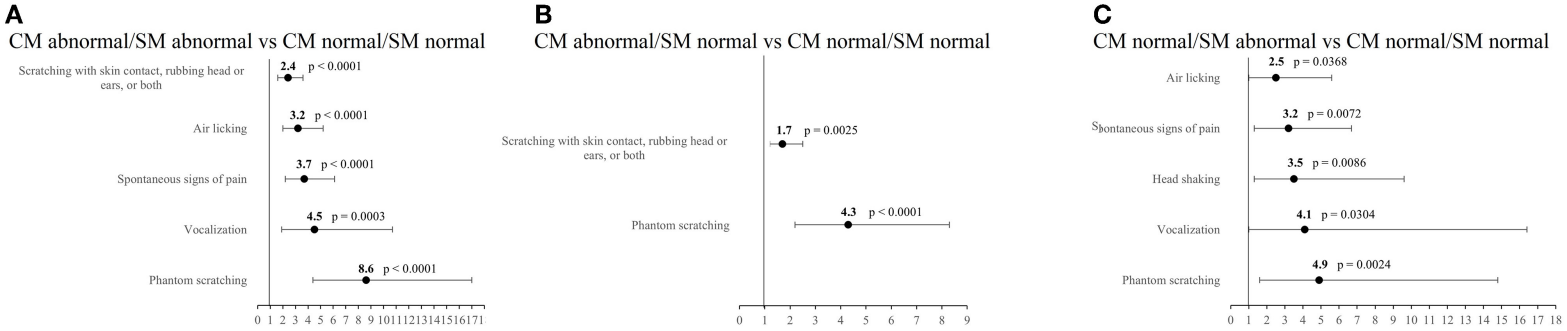
Odds ratios and 95% confidence intervals for specific owner-reported clinical signs. **(A)** CM abnormal/SM abnormal vs. CM normal/SM normal dogs, **(B)** CM abnormal/SM normal vs. CM normal/SM normal dogs, **(C)** CM normal/SM abnormal vs. CM normal/SM normal dogs.

Specific ORCS that occurred significantly more likely in one group vs. the other are listed here and depicted in Figure 4.

#### CM abnormal/SM abnormal dogs vs. CM normal/SM normal dogs

Five ORCS occurred significantly more often in CM abnormal/SM abnormal dogs compared to CM normal/SM normal dogs: (1) phantom scratching (OR 8.6, 95% CI 4.4–17.0, *p* < 0.0001), (2) vocalization (OR 4.5, 95% CI 1.9–10.7, *p* = 0.0003), (3) spontaneous signs of pain (OR 3.7, 95% CI 2.2–6.1, *p* < 0.0001), (4) air licking (OR 3.2, 95% CI 2.0-5.2, *p* < 0.0001), and (5) scratching with skin contact, rubbing head or ears, or both (OR 2.4, 95% CI 1.6–3.6, *p* < 0.0001).

#### CM abnormal/SM normal dogs vs. CM normal/SM normal dogs

Two ORCS occurred significantly more often in CM abnormal dogs compared to CM normal dogs: (1) phantom scratching (OR 4.3, 95% CI 2.2–8.3, *p* < 0.0001) and (2) scratching with skin contact, rubbing head or ears, or both (OR 1.7, 95% CI 1.2–2.5, *p* = 0.0025).

#### CM normal/SM abnormal dogs vs. CM normal/SM normal dogs

Five ORCS occurred significantly more often in CM normal/SM abnormal dogs compared to CM normal/SM normal dogs: (1) phantom scratching (OR 4.9, 95% CI 1.6–14.8, *p* = 0.0024), (2) vocalization (OR 4.1, 95% CI 1.0–16.4, *p* = 0.0304), (3) head shaking (OR 3.5, 95% CI 1.3–9.6, *p* = 0.0086), (4) spontaneous signs of pain (OR 3.2, 95% CI 1.3–6.7, *p* = 0.0072), and(5) air licking (OR 2.5, 95% CI 1.0-5.6, *p* = 0.0368).

### Quantitative syrinx measurements and ORCS

There were no significant differences in any of the measurements (STWR – T2W, STWR – T1W, SHRt – T2W, SHRt – T1W, SCSAR – T2W, SCSAR – T1W, SHRs – T2W, SHRs – T1W, SLC3R – T2W, and SLC3R – T1W) between SM abnormal dogs with ORCS and without ORCS. There were also no significant differences in any of the measurements for SM abnormal dogs with or without specific ORCS.

### Signalment and classification of CM and SM

#### Country of origin

Pomeranians originating from The Netherlands were 1.4 times more likely to be classified as CM abnormal than dogs from all other countries combined (OR 1.4, 95% CI 1.0–1.9, *p* = 0.0277).

There were no significant differences in SM classification for dogs grouped according to country of origin.

#### Pedigree

Dogs with a pedigree were not significantly more or less likely to be classified as CM abnormal than dogs without a pedigree. CM abnormal dogs without a pedigree were 1.9 times more likely to have a CM2 classification than CM abnormal dogs with a pedigree (OR 1.9, 95% CI 1.1–3.5, *p* = 0.0281).

There were no significant differences in SM classification for dogs grouped according to pedigree status.

#### Sex and neuter status

There were no significant differences in CM/SM classification for dogs grouped according to sex or neuter status.

#### Age

CM abnormal dogs (median 2.4 years, range 0.4–11.0) were significantly older than CM normal dogs (median 2.0 years, range 0.4–7.7) (*p* = 0.0036). Dogs aged ≥ 1.5 years were 1.5 times more likely to be classified as CM abnormal than dogs aged < 1.5 years (OR 1.5, 95% CI 1.1–2.0, *p* = 0.0144).

There was no significant age difference between CM1 and CM2 dogs.

SM abnormal dogs (median 2.9 years, range 0.8–11.0) were significantly older than SM normal dogs (median 2.1 years, range 0.4–10.6) (*p* < 0.0001). Dogs aged ≥ 1.5 years were 3.2 times more likely to be classified as SM abnormal than dogs aged < 1.5 years (OR 3.2, 95% CI 3.0–5.0, *p* < 0.0001).

There was no significant age difference between SM1 and SM2 dogs. There were no significant associations between any of the quantitative syrinx measurements and age.

#### Body weight

There were no significant associations between body weight and CM classification.

SM abnormal dogs (median 3.0 kg, range 1.4–6.0) weighed significantly less than SM normal dogs (median 3.4 kg, range 1.0–9.4) (*p* < 0.0001). Dogs with a body weight of < 2.5 kg were 6.3 times more likely to be classified as SM abnormal than dogs with a body weight of ≥2.5 kg (OR 6.2, 95% CI 3.9-10.2, *p* < 0.0001).

There was no significant difference in body weight between SM1 and SM2 dogs.

## Discussion

This is the first large study evaluating CM/SM in the Pomeranian dog breed. When interpreting the prevalence of CM/SM in Pomeranians in this study, it must be taken into account that included dogs were not sampled at random from a population of Pomeranians. Dog owners were mainly informed on the option of screening for CM/SM through social media platforms and breed clubs. But in contrast to other large studies that often describe screening results of dog intended for breeding, most of these Pomeranians were house-held dogs. And although some owners came specifically for diagnostic imaging because they noted ORCS, most dogs were presented for screening purposes. The prevalence ranges for CM and SM reported here for dogs without and with ORCS, were 53.4–62.4% and 11.9–34.7% respectively. If a random selection of Pomeranians from the population had been employed, the true prevalence would likely be included in these ranges. This is because both dogs with and without ORCS would be part of such a random selection procedure. From our prevalence percentages, it is clear that both CM and SM are prevalent in the Pomeranian dog breed, but that there is also a large proportion of dogs without either of these disorders. The latter subpopulation (CM normal/SM normal dogs) can be regarded as valid “controls” for studies focusing on possible genetic predispositions for CM and SM.

### Classification of CM/SM and ORCS

Both SM and CM were associated with the presence of ORCS. When the odds ratios for CM abnormal vs. CM normal and SM abnormal vs. SM normal were evaluated, the same 5 ORCS were identified to occur with increased likelihood in abnormal dogs: 1/phantom scratching, 2/vocalization, 3/scratching with skin contact, rubbing head or ears, or both, 4/spontaneous signs of pain, and 5/air licking. However, these uncombined classifications do not account for differences between dogs with or without one of both these disorders.

By combining the classifications (e.g. CM abnormal/SM normal) and comparing the groups against one another, it was evident that some specific ORCS were independently associated with each condition. We found that:

- phantom scratching and scratching with skin contact, rubbing head or ears, or both were associated with a CM abnormal classification (CM abnormal/SM normal vs. CM normal/SM normal dogs);
- phantom scratching, vocalization, head shaking, spontaneous signs of pain, and air licking were associated with a SM abnormal classification (CM normal/SM abnormal vs. CM normal/SM normal dogs).

Whilst for some of these ORCS the likelihood of their presence was increased by having both CM and SM, this was not the case for all ORCS that were associated with either CM or SM. For instance, CM abnormal/SM abnormal dogs were 8.6 times more likely to be reported to show phantom scratching by their owners than CM normal/SM normal dogs. When CM abnormal/SM normal dogs and CM normal/SM abnormal dogs were compared to this same control group of CM normal/SM normal dogs, odds ratios of 4.3 and 4.9 were found, respectively. Therefore, there seems to be an additive effect of having both CM and SM on the likelihood of owners reporting this sign. Finding significant associations between the presence of CM and ORCS provides further support for the existence of CM associated pain (CM-P) (4, 10, 11, 18, 19).

Phantom scratching has been reported to be a specific clinical sign for SM, with one study in the CKCS reporting that phantom scratching occurred in 0% of dogs without SM (19). Another study could not find a statistically significant relationship between the presence of owner-reported scratching (yes or no) and the presence of SM (24). This latter study did not specifically differentiate between scratching with or without skin contact. It is possible that owners misinterpret scratching for phantom scratching and *vice versa*. Still, based on our findings, it would be prudent not to consider phantom scratching as a pathognomonic ORCS of SM. Rather, the presence of phantom scratching could be regarded as a reliable indicator that either one or both of the disorders is or are present. There is some evidence that spinal cord changes related to syrinx formation and asymmetry of SM is related to phantom scratching or scratching (20, 24, 25). Such reports also delve into the pathophysiology of phantom scratching (i.e., spinal cord pathways involved in the scratch reflex) and hypothesize the influence of SM on its occurrence. How the ORCS of phantom scratching in Pomeranians or other dogs with CM but without SM fits into these hypotheses remains to be determined.

Spontaneous signs of pain as an ORCS were significantly associated with the presence of SM in this study. Several signs observed by the owners are in line with the study of Sparks et al. (25). Examples noted in that study were “crying out”, “head shy”, “side pain”, “running from pain”, “neck pain”, “exercise induced pain”, “woken by pain”, and “collar sensitivity” as owner-reported pain signs (25). Additional signs the owners noted were “licking limb/paw”, “squinting/avoiding light”, and “touch or grooming aversion”. These clinical and behavioral signs have been noted in a recent study by Rusbridge et al. (19). “Pain” is defined by the International Association for the Study of Pain (IASP) as an unpleasant sensory and emotional experience associated with, or resembling that associated with, actual or potential tissue damage (26). What is or is not associated with pain in animals, is therefore always a subjective interpretation. Studies on quantitative assessments of SM-related neuropathic pain have yielded conflicting results (27–29). Taking this into account, including whether or not signs of pain where provoked vs. spontaneous seemed to be an interesting differentiation to investigate in our study population. And indeed, we found a significant association for spontaneous signs of pain with SM and no significant association for provoked signs of pain. So, regardless of which specific sign of pain is observed by an owner, whether it is spontaneous or provoked, it can be valuable for assessing whether a dog is likely to have CM/SM or not.

Associations between lateralization of SM (i.e., unilateral extension of a syrinx into the dorsal horn gray matter) and the occurrence of neuropathic pain signs (including phantom scratching) have been reported (20, 25). In our study, we did not identify an association between ORCS and asymmetry of SM. This may be due to a low number of Pomeranians showing lateralization of SM classified as SM2 in our study [39/190 dogs (20.5%)]. Lateralization of ORCS was not accounted for in this study. Therefore, we were unable to assess if lateralization of SM was associated with lateralization of ORCS as has been reported in the CKCS (25).

Regarding localization of SM in Pomeranians, we found that 59.5% of dogs with SM had either extensive (both cervical and thoracic, continuous) or multifocal (both cervical and thoracic, discontinuous) syrinx formation. This finding is in line with results from studies in the CKCS, where SM is found in other than a cervical spinal cord location in many instances (30, 31). As opposed to CKCS, the thoracic spinal cord was most severely affected in most cases of extensive and multifocal localizations. The MRI scans were made up to at least T4, but inclusion of the whole spinal cord was not a requirement for this study. Therefore, we are unable to assess the exact distribution of SM in these dogs. Nevertheless, it is evident that SM in Pomeranians affects not only the cervical spinal cord but more caudal spinal cord segments as well.

Of note, many of the behaviors witnessed by owners may be regarded or classified as undesirable behaviors. A recent study found dogs aged < 3 years of several breeds to be of increased risk of death (mostly by euthanasia) due to undesirable behavior (32). Among these breeds were the CKCS and the Chihuahua. The number of Pomeranians in that study was low and no significantly different risk was identified for that breed compared to Labrador Retrievers. However, as ORCS were reported in 52.5% of Pomeranians in this study and many of these reflect behavioral signs that may be regarded as undesirable behaviors, it is possible that Pomeranians, like the CKCS and Chihuahua, are at increased risk of death by euthanasia related to those behaviors. In other words, owners that notice such signs in their dogs may ultimately elect euthanasia (21, 32, 33). An unknown portion of the owners of the included Pomeranians may have accidently misinterpreted their dog's behavior as indicative for CM and/or SM. The authors therefore stress that MRI is needed to differentiate between ORCS associated with CM an/or SM and behavioral problems.

Another consideration with respect to ORCS is the fact that ORCS may be related to other pathology. For instance, in the CKCS, clinical signs related to primary secretory otitis media (PSOM) may mimic those of CM/SM and this disorder may be present concomitantly (34, 35). PSOM (also called middle ear effusion or otitis media with effusion) has been reported in other brachycephalic breeds (e.g., French bulldog) as well (36–38). While assessment of the prevalence of PSOM was not an aim of this study, no formal diagnosis of PSOM was made in any of the included Pomeranians in this study. Thus, the prevalence of PSOM in the Pomeranian is likely to be very low. This may reflect the differences in skull conformation between CKCS and brachycephalic breeds (e.g., bulldogs, pugs, and boxers) reported to be affected by PSOM so far.

### Quantitative syrinx measurements and ORCS

Statistical analysis did not reveal any significant associations between quantitative syrinx measurements and ORCS. This hypothesis was accepted, as statistical analysis did not reveal any significant associations between the presence or absence of ORCS and quantitative syrinx measurements in dogs with SM. There are conflicting results in literature with regard to associations of clinical signs with quantitative measurements or semiquantitative classifications of SM severity in dogs. Some studies have found associations between the severity of SM and the presence of (owner-reported) clinical signs (19, 30, 31), while others have found no such association (25).

We included various types of quantitative assessments in our study, with each measurement being based on T2W and T1W MR images. We incorporated both T1W- and T2W-based measurements, as previous studies have variably utilized either T1W- or T2W-based measurements (20, 25, 30, 39–41). All T1W-based measurements were statistically significantly smaller than T2W-based measurements in this study. Based on this finding, comparing outcomes from studies that use T2W measurements compared to T1W measurements is not reliable. Differentiation between true syrinx margins and spinal cord oedema surrounding the syrinx is more difficult on T2W- than T1W-images (42). Indeed, this was one reason why previous researchers preferred T1W-images (29). The findings of our study support this. However, we did find that T2W-based and T1W-based measurements were all very strongly correlated. So, studies that also use T2W-based measurements are not necessarily invalid.

Several previous studies and currently employed CM/SM grading schemes for the CKCS measured or measure SM parameters absolutely (e.g., a syrinx diameter of 2.2 mm), or classify SM in groups, or classify dogs as having SM or not based on size (e.g., >2 mm) (18–20, 27–29, 39–41, 43, 44 and see text footnote 1). Fewer studies aimed to provide relative syrinx sizes, providing a ratio between a syrinx parameter and another parameter (e.g., syrinx height: C3 vertebral depth or the spinal cord itself) (25, 30, 43). One must keep in mind though that when absolute measurements are used rather that relative measurements and dogs are categorized (f.i. < 2 mm syrinx width vs. >2 mm syrinx width), dogs that may fall into one category based on T2W-measurements may fall into another category based on T1W-measurements (39, 40). As syrinx size is a continues variable, it does not seem logical to categorize dogs according to the measurements. In the authors' opinion, it is more logical to view dilatation of the central canal or presence of SM as abnormal and thus categorize dogs as SM normal and SM abnormal as we did in this study.

Body size varies between individual dogs and so does the diameter of the spinal cord between different locations (e.g. intumescence vs. non-intumescence). This makes using a vertebra as a reference less logical, as the ratio will be inherently lower when a syrinx is measured in a non-intumescence compared to an intumescence segment of the spinal cord. So, rather than reporting absolute measurements of SM parameters, we report ratios of these measurements compared to the spinal cord itself (for height, width, cross-sectional area) as a reference (25). The C3 vertebral length was used as a reference to assess syrinx length.

### Signalment factors and CM/SM classification

When compared to all other countries combined, dogs from The Netherlands were slightly more likely (OR 1.4) to have CM than dogs from other countries. However, Dutch Pomeranians are mostly bred using Russian ancestors combined with imports from countries such as America and Germany. There was no difference when specifically comparing dogs from The Netherlands vs. dogs from Russia, suggesting that there likely is no real difference between prevalence of CM between those countries. From studies in other breeds like the CKCS, it is clear that the prevalence of CM/SM varies between studied populations (1, 3, 8, 9, 12–17).

Having a pedigree (i.e., being purebred with appropriate documentation) was not associated with increased or decreased likelihood of having CM/SM. However, CM abnormal dogs without a pedigree were more likely (OR 1.9) to have a CM2 classification than CM abnormal dogs with a pedigree. It is possible that the Pomeranians without a pedigree are the offspring of Dutch Pomeranians with a pedigree. However, this is speculative as in most dogs without a pedigree the exact origin could not be ascertained. Possibly, pure-bred breeders select their breeding stock more securely and are able to decrease the prevalence of CM in their stock. Future studies may provide more evidence to support the effectiveness of screening and selection for CM/SM in Pomeranians, as has been evaluated for CKCS (14, 17).

Age was significantly associated with CM as well as SM classification. Dogs aged < 1.5 were less likely to have CM/SM than dogs aged ≥1.5 years. This has implications for selection of dogs for breeding purposes based on screening procedures. Dogs that are classified as CM/SM normal at an early age may be classified as CM/SM abnormal at a later age. Repeated screenings or single screening at an advanced age could be considered to overcome the effect of age on screening outcomes (14, 18). A similar observation was made in the CKCS. Knowler et al. concluded in 2011 that the most effective strategy would be to use only dogs that are scanned after the age of 5 (14). But these findings seem to suggest that CM/SM is, like in the CKCS, a so-called late onset disease. Such an association may be explained by skull conformation, maturation, and changes in craniocerebral morphology and syringomyelia already documented in several other studies for the CKCS (8, 14, 15, 17, 30, 41, 44–47). There are currently no specific studies that document objective information on skull maturation or craniocerebral morphology changes in the Pomeranian. Although the classification of SM was associated with age, the quantitative syrinx parameters were not. Other studies have identified changes in syrinx size with advancing age, longitudinally (41, 43). As this study did not longitudinally assess quantitative syrinx parameters within individuals over time, an effect of age on quantitative syrinx parameters cannot be excluded and requires future study.

There was no association between body weight and CM classification, but dogs with SM weighed significantly less than dogs without SM. We did not account for body condition score in this study, so body weight would not necessarily reflect size perfectly in this study. Still, it is safe to conclude that smaller dogs had an increased risk of being affected by SM than larger dogs. No association was found for CM/SM and body weight in other breeds such as the Chihuahua and CKCS, though not all studies tested for associations with body weight (8, 18, 19, 29, 40). The influence of body weight or other parameters accounting for size differences between dogs deserves more attention in future studies. Selection of larger dogs may be one factor of use for breeders in selecting dogs less likely to give offspring with SM.

Limitations to this study include the use of both low- and high-field MRI scanners to acquire MRI studies, the lack of full neurological examinations for each included dog, and lack of inclusion of the whole spinal cord in studies to assess the extent of syringomyelia (as discussed above). Also, as mentioned earlier in this discussion, the Pomeranians included in this study were not selected at random. Currently, there are no specific studies that evaluate low- vs. high-field MRI studies for the diagnosis of CM/SM. High-field MRI studies, however, are considered the gold standard for diagnosing CM/SM in dogs (1, 10). Low-field MRI studies may yield low quality images and influence assessment and measurements. Although MRI studies with artifacts or insufficient image quality that did not allow for accurate assessments or measurements were excluded in this study, we cannot rule out the possibility of misclassification for dogs scanned with low-field MRI machines. Importantly though, the use of high-field vs. low-field MRI machines for image acquisition does not by definition guarantee better image quality, since image quality is not determined solely by field strength. Among the strong points on this study are the large number of dogs that could be included and the fact that most Pomeranians were house-held dogs both with and without ORCS.

In conclusion, this study documents several associations for CM/SM classification and ORCS and signalment factors. Veterinary clinicians may use these findings to increase the likelihood of correctly determining the presence or absence of CM/SM in Pomeranians, though the diagnosis itself still necessitates diagnostic imaging (MRI). Breeders may consider using the information regarding signalment factors as well as ORCS associated with CM/SM classifications to select dogs for screening procedures. But an MRI-based diagnosis is needed to properly ascertain the exact CM/SM status of their breeding stock until a fool-proof characteristic or genetic marker is found.

## Data availability statement

The raw data supporting the conclusions of this article will be made available by the authors, without undue reservation.

## Ethics statement

The animal studies were approved by Animal Welfare Body Utrecht, Utrecht University, The Netherlands. The studies were conducted in accordance with the local legislation and institutional requirements. Written informed consent was obtained from the owners for the participation of their animals in this study.

## Author contributions

KS: Conceptualization, Data curation, Formal analysis, Funding acquisition, Investigation, Methodology, Resources, Visualization, Writing—original draft, Writing—review & editing. IC: Conceptualization, Methodology, Supervision, Writing—review & editing. KB: Data curation, Investigation, Resources, Writing—review & editing. PM: Conceptualization, Data curation, Funding acquisition, Investigation, Methodology, Project administration, Resources, Supervision, Validation, Writing—review & editing.

## Footnotes

^1^Adapted from the BVA/KC scheme for CM grading Chiari Malformation/Syringomyelia Scheme (CM/SM Scheme) [Online] (2023). Available online at: https://www.bva.co.uk/canine-health-schemes/cmsm-scheme/ (accessed August 17, 2023).
